# CD109在初发急性髓系白血病患者中的表达特征及临床意义

**DOI:** 10.3760/cma.j.issn.0253-2727.2023.09.012

**Published:** 2023-09

**Authors:** 丽霞 王, 仪 陈, 韶彤 董, 方刚 任, 耀方 张, 建梅 常, 艳红 覃, 秀花 陈, 宏伟 王, 智芳 徐

**Affiliations:** 山西医科大学第二医院血液科，血液病分子诊疗山西省重点实验室，太原 030001 Department of Haematology, The Second Hospital of Shanxi Medical University, Shanxi Provincial Key Laboratory of Molecular Diagnosis and Treatment of Hematological Diseases, Taiyuan 030001, China

膜蛋白CD109属于GPI联结糖蛋白，参与多种重要信号通路，如TGF-β/Smads信号通路[1]–[2]、JAK-STAT信号通路[3]–[4]和表皮生长因子受体通路[5]–[6]。CD109最初是在急性髓系白血病（AML）细胞系KG-1a鉴定的细胞表面抗原[7]，主要表达于胎儿和成人CD34阳性的骨髓单个核细胞亚群、激活的T淋巴细胞、激活的血小板、白血病原巨核细胞、内皮细胞、间充质干细胞亚群和一些人类肿瘤细胞系中[8]，但不表达于人类静止的T细胞、血小板或外周血白细胞[9]。本研究探索了CD109在初发AML（M_3_除外）患者中的表达特征以及与CD34表达的相关性。

## 对象与方法

1. 研究对象：收集2019年1月至2021年1月山西医科大学第二医院血液科确诊的75例AML患者（除外PML-RARα阳性患者）初诊时骨髓标本用于荧光定量PCR（RT-qPCR）分析，其中男48例，女27例，中位年龄52（12～81）岁，收集17名正常人［男10名，女7名，中位年龄46（33～66）岁］的骨髓标本作为对照。100例AML患者的新鲜骨髓标本用于流式细胞术分析。本研究获得本单位伦理委员会审查批准［批准号：（2020）YX第（069）号］。诊断依据2016版WHO标准[10]，根据NCCN指南（2018年版）基于细胞遗传学和分子遗传学将AML患者进行危险分层。收集AML患者临床基本资料和实验室相关指标，包括性别，年龄，初诊时WBC、PLT、HGB、骨髓原始细胞比例，染色体核型，免疫分型，基因突变等。对CD34阳性AML患者进行了随访，随访截止时间为2023年1月。人AML细胞株KG-1a由中国医学科学院血液病研究所馈赠，人急性原粒细胞白血病细胞株Kasumi-1、人急性单核细胞白血病细胞株THP-1、人原巨核细胞白血病细胞株UT-7、人慢性髓性白血病细胞株K562来自山西医科大学第二医院血液科实验室。

2. RNA提取和cDNA的合成：根据试剂厂商提供的方法使用Trizol（美国Invitrogen公司产品）从1×10^6^个骨髓单个核细胞中提取总RNA。用多功能微孔板（美国Biotek公司cytation3型）测定分离的总RNA的浓度和纯度。*A*_260_/*A*_280_在1.8～2.0为合适的RNA纯度。使用PrimeScript™ RT试剂和gDNA Eraser（日本TaKaRa公司产品）按照生产厂商提供的操作步骤合成互补DNA（cDNA）。

3. RT-qPCR：CD109引物序列和内参基因GAPDH引物序列如下：CD109上游5′-TAGCAGTCCACATGTCCGAAAGCA-3′，下游5′-AACCAGTAGCCACCCAAGAAGTGA-3′，GAPDH上游5′-GAAGGTGAAGGTCGGAGTC-3′，下游5′-GAAGATGGTGATGGGATTTC-3′。反应条件（7300荧光实时定量PCR扩增仪，美国Applied Biosystems公司产品）：95 °C预变性30 s，随后95 °C变性10 s，61 °C退火30 s，72 °C延伸31 s，共40个循环。采用ΔΔCt法计算某样本的目的基因mRNA相对表达量（RQ），RQ＝2^−ΔΔCt^，每一样本均设2个复孔，计算其均值作为RQ值。每次PCR后均进行熔解曲线分析以确认扩增产物的特异性。

4. 流式细胞术：采用流式细胞术进行免疫表型分析，运用Kaluza 2.0（美国Beckman Coulter公司产品）软件进行分析。AML患者中的原始细胞通过CD45/SSC进行设门。涉及CD109和CD34的抗体组合为：CD38-FITC/CD109-PE/CD34-PC5/CD45-PC7，CD109表达的测量方法：①阳性表达率（gate％）：以相应的同型抗体标记的细胞的荧光分布直方图作为阴性对照。②平均荧光强度（MFI）：以CD109 MFI与相应荧光染料的同型对照MFI的比值进行计算。

5. 细胞培养：KG-1a细胞系培养于含10％胎牛血清的IMDM培养基中，37°C、5％CO_2_、饱和湿度的培养箱中培养。Kasumi-1、THP-1、UT-7、K562细胞系于含10％胎牛血清的RPMI 1640培养液培养，使用对数生长期的细胞进行后续实验。

6. 统计学处理：采用SPSS 25.0统计软件和GraphPad Prism 5.01统计软件进行数据分析和图形处理。连续变量的正态性判定采用Shapiro-Wilk检验，若为正态分布则用均值±标准差表示，两组间比较采用Student *t*检验。若为非正态分布则用*M*（*IQR*）表示，采用Mann-Whitney *U*检验，多组间比较采用Kruskal-Wallis秩和检验，当组间总的差异有统计学意义，进一步采用Dunn法进行多重比较。两个变量因素的相关密切程度采用相关性分析。已分类的变量显示为百分比，采用卡方检验（N>40，理论频数≥5的格子≥20％）或Fisher确切概率法进行比较。生存曲线分析采用Kaplan-Meier法。所有报告的检验均为双尾检验，*P*<0.05为差异具有统计学意义。

## 结果

一、CD109 mRNA在AML患者中的表达

CD109 mRNA在AML患者中呈异质性表达。AML组与正常对照组中位表达水平分别为2.656（0.714～8.268）和0.815（0.635～1.582），差异有统计学意义（*P*<0.05），由于AML的异质性较强，我们进一步研究CD109 mRNA在AML各亚型中的表达，结果发现在M_0_、M_1_、M_4_组明显高于正常对照组（*P*值均<0.05）。而在M_2_、M_5_、AML伴重现性遗传学异常组中，CD109 mRNA的表达水平与正常对照组相比差异均无统计学意义（*P*值均>0.05）（图1）。

**图1:**
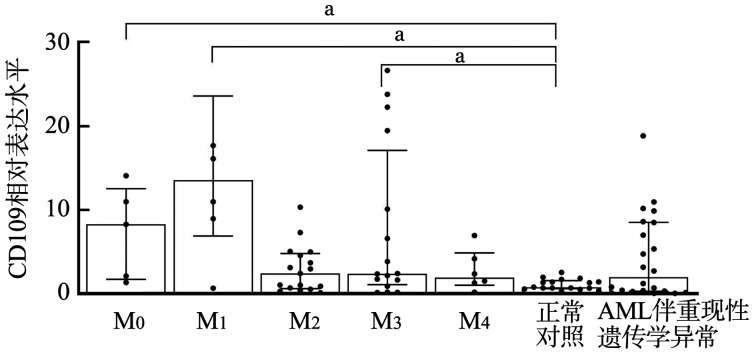
CD109 mRNA在不同分型急性髓系白血病（AML）患者的相对表达水平（^a^*P*<0.05）

二、CD109 mRNA在CD34阳性和CD34阴性AML患者中的表达差异

1. CD109 mRNA在CD34阳性和CD34阴性AML患者中的表达：流式细胞术检测CD34阳性的cut off值设定为≥20％。结果发现CD109 mRNA的表达水平在CD34阳性组［2.976（0.636～8.785）］明显高于CD34阴性组［0.899（0.274～2.411）］和正常对照组［0.815（0.636～1.582）］（*P*值均<0.05），而后两者差异无统计学意义（*P*>0.05）。

2. CD109 mRNA分别在CD34阳性和CD34阴性AML组中与融合基因RUNX1-RUNX1T1、CBFβ-MYH11以及FLT3-ITD基因突变的相关性：在CD34阳性组中，CD109 mRNA在FLT3-ITD突变型患者中的表达明显高于FLT3-ITD野生型患者（*P*<0.01）；而在CD34阴性组中，CD109 mRNA表达水平与融合基因RUNX1-RUNX1T1、CBFβ-MYH11及FLT3-ITD基因突变无关（*P*>0.05）（表1）。

**表1:** CD109 mRNA在CD34阳性、CD34阴性急性髓系白血病患者中与融合基因及核型分层的相关性不同分组间的比较

组别	CD34阳性患者		CD34阴性患者	
	例数	表达水平	例数	表达水平
RUNX1-RUNX1T1				
阳性	4	1.239（0.048~4.052）	5	0.405（0.118~2.063）
阴性	22	4.428（0.928~10.48）	8	0.727（0.235~2.149）
CBFβ-MYH11				
阳性	5	3.182（1.460~8.976）	3	1.224（0.924~1.304）
阴性	22	4.428（0.923~10.48）	8	0.727（0.235~20149）
FLT3-ITD突变				
阳性	8	10.210（7.047~23.38）^b^	9	0.924（0.360~3.879）
阴性	39	1.648（0.563~8.268）	21	0.616（0.223~2.406）
染色体核型预后				
低危	7	0.295（0.051~2.745）	4	0.285（0.092~0.409）
中危	36	3.493（0.684~9.721）^a^	20	1.378（0.252~3.393）^a^
高危	4	6.978（2.486~12.79）^a^	3	0.530（0.108~0.616）

**注** 与阴性或低危组比较，^a^*P*<0.05，^b^*P*<0.01；染色体核型预后低危指t（8; 21），t（15; 17）或inv（16）；高危指3q，−5或del（5q），−7，t（9; 22），复杂（≥3个异常）；中危指正常核型，+ 8，+ 21，+ 22，del（9q），11q23异常或其他异常（良好或不良风险组除外）[11]

3. CD109 mRNA在CD34阳性和CD34阴性AML患者中不同的核型危险度分组中的表达：在CD34阳性患者中，预后高危组和中危组CD109 mRNA水平明显高于预后低危组（*P*≤0.05）。在CD34阴性患者中，预后中危组CD109 mRNA水平明显高于预后低危组（*P*<0.05）（表1）。

三、CD109 mRNA表达与CD34阳性AML患者临床及实验室特征的关系

依据CD109 mRNA在AML患者中的中位表达水平2.745，将53例AML患者分为CD109低表达组（26例）和CD109高表达组（27例）。结果表明，CD109低表达组与高表达组在NCCN危险分层、核型危险度、FLT3-ITD基因突变的分布上差异有统计学意义（*P*值均<0.05），而WBC、HGB、PLT、原始细胞比例、2016 WHO分类、诱导后的完全缓解率差异均无统计学意义（*P*值均>0.05）（表2）。

**表2:** CD34阳性急性髓系白血病（AML）患者中CD109高表达组与低表达组的临床特征比较

临床特征	CD109低表达组（26例）	CD109高表达组（27例）	*P*值
性别［例（%）］			0.695
男	15（58）	17（63）	
女	11（42）	10（37）	
年龄［岁，*M*（范围）］	46（19~77）	52（17~81）	0.981
WBC［×10^9^/L，*M*（范围）］	9.8（1.2~376）	24（0.5~198）	0.237
PLT［×10^9^/L，*M*（范围）］	30.5（5~619）	27（3~418）	0.650
HGB［g/L，*M*（范围）］	86（41~126）	78（11~118）	0.086
骨髓原始细胞［%，*M*（范围）］	55（3.5~93）	60.8（22~97）	0.901
WHO 2016分类［例（%）］			0.313
M_0_	2(7)	1(4)	
M_1_	2(8)	2(7)	
M_2_	7(27)	3(11)	
M_4_	1(4)	4(15)	
M_5_	1(4)	2(7)	
M_6_	0(0)	0(0)	
AML伴重现性遗传学异常［例（%）］	13(50)	12(45)	
AML伴骨髓增生异常相关改变［例（%）］	0(0)	3(11)	
NCCN危险分层［例（%）］			0.003
低危	7(27)	1(4)	
中危	18(69)	21(77)	
高危	1(4)	5(19)	
核型预后［例（%）］			0.012
低危	23(88)	16(59)	
中危	2(7)	6(22)	
高危	1(5)	5(19)	
FLT3-ITD基因［例（%）］			0.023
突变型	0(0)	6(22)	
野生型	26(100)	21(78)	
诱导后完全缓解^a^［例（%）］			0.780
是	9(35)	11(41)	
否	17(65)	16(59)	

**注** ^a^诱导治疗第25～35天评估治疗反应达完全缓解（CR）或血细胞未完全恢复的CR

四、流式细胞术检测AML患者原代细胞中CD109蛋白的表达及其与CD34表达的关系

1. CD109蛋白的表达率及其与CD34表达的相关性分析：我们统计了CD109在AML患者（100例）中的阳性表达率，当cut off值设定为20％时，阳性患者为58例（58％），设定为10％时，阳性患者为72例（72％）。将cut off值设定为10％，进一步研究了CD109与CD34在AML患者原始细胞中表达的相关性，结果发现CD109与CD34的表达率呈正相关（*R*^2^＝0.076 8，*P*<0.05），而表达强度没有相关性（*R*^2^＝0.000 3，*P*>0.05）。

2. CD109蛋白在CD34阳性与CD34阴性原始细胞中表达的比较：在检测的100例AML患者中，CD34阳性患者为77例，阴性患者为23例。结果表明CD109蛋白在CD34^+^AML患者中的阳性表达率［44.62％（13.99％～79.59％）］明显高于CD34^−^AML患者［20.96％（2.87％～48.71％）］（*P*<0.05），而MFI差异无统计学意义（*P*>0.05）。我们进一步比较了CD109在CD34阳性患者中的CD34阳性原始细胞与CD34阴性原始细胞的阳性表达率和MFI，结果发现CD109蛋白在CD34^+^原始细胞中的表达［44.62％（13.99％～79.59％）］显著高于CD34^−^的原始细胞［16.28％（5.62％～41.66％）］（*P*<0.001），而MFI差异无统计学意义（*P*>0.05）。

五、CD109 mRNA表达水平对CD34阳性AML患者生存的影响

将53例CD34阳性的AML患者分为CD109高表达组（27例）和CD109低表达组（26例），分析两组患者的总生存（OS）期和无进展生存（PFS）期。结果显示CD109低表达组的OS期明显长于CD109高表达组（*P*<0.01），而PFS期差异无统计学意义（*P*>0.05）（图2A、B）。

**图2:**
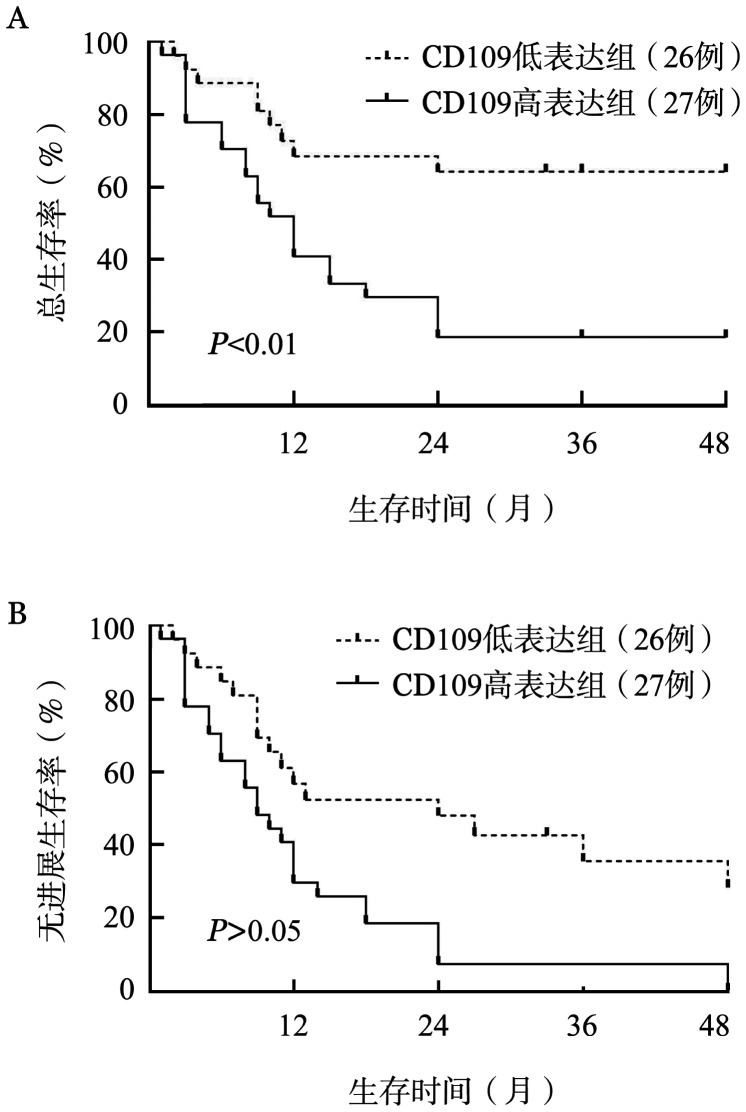
CD109低表达组与CD109高表达组的总生存（A）、无进展生存（B）曲线

六、CD109 mRNA及蛋白水平在髓系白血病细胞系中的表达

CD109 mRNA和蛋白在不同髓系白血病细胞系中的表达见图3A、3B，以CML K562细胞为阴性对照，CD109在KG-1a和Kasumi-1细胞系中的表达显著增高（*P*值均<0.05）；在THP-1、UT-7细胞系表达降低（*P*值均>0.05）。

**图3:**
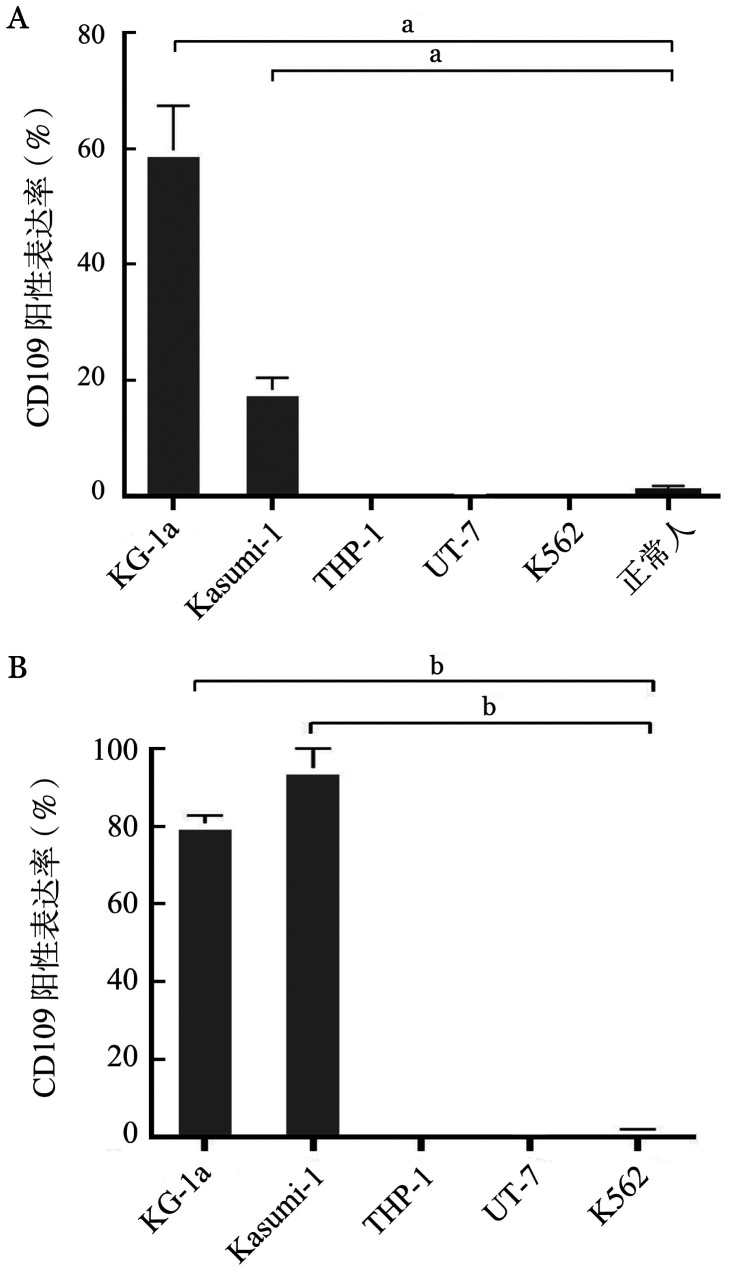
CD109在髓系白血病细胞系中的表达 **A** 荧光定量PCR检测CD109 mRNA的表达水平；**B** 流式细胞术检测CD109蛋白的表达水平（^a^*P*<0.01，^b^*P*<0.05）

## 讨论

CD109是近年来发现的一种细胞表面抗原，属于糖基化磷脂酰肌醇（GPI）联结的糖蛋白[12]。研究发现，CD109的异常表达与肺腺癌疾病[4]、胶质母细胞瘤[6]、弥漫大B细胞淋巴瘤[9]等多种恶性疾病的预后不良相关。也有研究表明CD109在AML基因表达谱中表达上调并与AML的预后相关，是其预后不良的指征[13]–[16]。但以上结论均是基于数据库高通量分析得出。Deepak Shyl等[17]首次报道了CD109和LRP12可能作为AML诊断意义的生物标志物。关于CD109在AML患者中的表达特征尚不清楚。所以我们研究了CD109在AML尤其在CD34阳性AML患者中的表达及其与患者临床特征的相关性，并在AML患者原代细胞中利用多参数流式细胞术研究CD109与CD34抗原表达的关系。

我们比较了初诊AML患者与正常人骨髓细胞中CD109 mRNA的表达水平，二者差异有统计学意义，由于AML是一种异质性很高的疾病，因此我们进一步将AML患者依据WHO分型进行分组，结果发现与正常人相比，CD109在M_0_、M_1_、M_4_组的表达水平明显升高，对这一结果可能的解释是CD109在表达CD34的造血干细胞上表达最强[18]，而M_0_、M_1_处于AML分化的早期阶段，CD34抗原表达较高。

鉴于此，依据CD34抗原表达情况，将AML患者分为CD34阳性组和CD34阴性组，结果发现CD109在CD34阳性组中的表达明显高于正常对照组和CD34阴性组，而CD34阴性组与正常对照组差异无统计学意义，表明AML患者中CD109抗原与CD34抗原之间可能存在相关性。进一步分析在CD34阳性的AML患者中，CD109在FLT3-ITD基因突变阳性组表达明显升高，有研究表明FLT3-ITD突变和CD34的联合表达是AML患者预后不良的重要预测因素[19]，而我们的研究结果表明CD109在CD34阳性AML患者FLT3-ITD阳性组中表达明显增高，所以推测CD109可能与AML的预后有关。同时在CD34阳性AML患者组中，核型预后分组中低危组CD109的表达明显低于高危组和中危组。我们同时也比较了CD109与CD34阳性的AML患者的临床特征关系，结果发现CD109高表达组与低表达组在NCCN危险分层、核型预后和FLT3-ITD基因突变的分布也有统计学差异。因此，提示CD109可能与CD34阳性AML患者的不良预后有关。此外，也有研究利用人工神经网络（ANN）的机器学习方法报道了CD109的基因表达谱与AML患者的不良预后相关[15]–[16]，这与我们的研究结果是一致的。

同时利用流式细胞术研究CD109与CD34抗原的表达关系，结果表明在蛋白水平上，CD109与CD34在AML患者原代细胞中的表达呈正相关，与前述mRNA水平的研究一致。通过检测CD34阳性和CD34阴性AML患者中CD109的表达水平，结果表明CD109膜蛋白在CD34阳性患者中的阳性表达率明显高于CD34阴性患者。而且在CD34阳性的患者中，CD109在CD34阳性原始细胞中的表达率显著高于CD34阴性原始细胞。这与Giesert[8]和Hwang等[18]的研究结果一致。此外，在髓系白血病细胞系中，无论是在mRNA水平还是蛋白水平上，CD109在CD34阳性细胞系KG-1a和Kasumi-1中的表达均显著增高，进一步提示在AML中CD109与CD34之间存在一定的相关性。

鉴于此，我们将CD34阳性AML患者分为CD109高表达组和CD109低表达组进行生存分析，结果显示CD109高表达组的OS期显著短于CD109低表达组。有研究报道CD109在中危以及高危AML亚组的基因谱中的表达是上调的[20]–[21]。因此推测CD109可能会成为预后不良AML的分子靶标。

综上所述，CD109在初发AML患者中表达增高并且呈异质性表达。在CD34阳性AML中，CD109的表达与FLT3-ITD基因突变，核型预后分组有关，且CD109高表达组的OS期显著短于CD109低表达组。由于AML是一种异质性很强的疾病，且CD109与CD34的表达密切相关，所以CD109可能会成为AML患者的分子治疗靶点。有关CD109能否指导AML疾病的预后以及其参与AML的发生机制还有待进一步研究。
